# Is the effect of antihypertensive drug therapy on blood pressure control and prevalent cardiovascular disease associated with dietary sodium-potassium ratio? A cross-sectional study based on NHANES

**DOI:** 10.3389/fcvm.2026.1763612

**Published:** 2026-03-23

**Authors:** Ou Zhang, Yintang Wang, Yu Geng, Lei Bi, Boqun Shi, Yuhao Zhao, Xiaofan Wu

**Affiliations:** 1 Department of Cardiology, Beijing Anzhen Hospital, Capital Medical University, Beijing, China; 2 Department of Cardiology, Beijing Tsinghua Changgung Hospital, School of Clinical Medicine, Tsinghua University, Beijing, China

**Keywords:** antihypertensive therapy, blood pressure control, cardiovascular disease, endothelial injury, hypertension, sodium-to-potassium ratio

## Abstract

**Objective:**

To evaluate whether antihypertensive treatment strategy and dietary sodium-to-potassium (Na/K) ratio are associated with blood pressure (BP) control and cardiovascular disease (CVD), and whether Na/K interacts with treatment effects.

**Methods:**

We analyzed 4,800 treated hypertensive adults (BP control defined as <130/80 mmHg) from NHANES 2009-2018 using survey-weighted logistic models and interaction analyses. Parallel endothelial experiments were performed in HUVECs exposed to normal or high Na/K, Ang II, and antihypertensive drugs.

**Results:**

Combination therapy was not associated with improved BP control (OR = 0.89; *P* = 0.214) but was associated with higher CVD prevalence (OR = 2.40; *P* < 0.001) compared with monotherapy. The Na/K ratio showed no significant association with BP control or CVD across quartiles, and no meaningful interaction with treatment strategy was observed on multiplicative or additive scales. In cell experiments, high Na/K conditions intensified Ang II-induced endothelial injury, while the protective effects of losartan and combination therapy remained comparable under both sodium conditions.

**Conclusions:**

Pharmacologic treatment intensity and dietary Na/K ratio appeared to influence vascular outcomes independently in treated hypertension. Critically, the lack of dietary Na/K effect modification in the clinical population is biologically explained by the maintained drug efficacy under high Na/K conditions.

## 1 Introduction

As one of the most widespread and adjustable risk contributors to cardiovascular disease (CVD), hypertension affects over 1.2 billion adults and contributing to more than 10 million each year through mechanisms involving myocardial infarction and heart failure (1–3). Strict blood pressure (BP) is imperative to reduce the prevalence of significant composite CVD, including myocardial infarction, heart failure, and cerebrovascular accidents (4). Although the use of antihypertensive medications is a well-established strategy for BP management, there remains ongoing debate regarding the relative benefits and risks of monotherapy compared with combination therapy (5). While combination regimens are often prescribed to enhance treatment efficacy, concerns about overtreatment, increased pill burden, and potential side effects underscore the need for careful evaluation of their clinical outcomes.

Dietary factors have increasingly been recognized as important contributors to cardiovascular health (6). Among them, the dietary sodium-to-potassium (Na/K) ratio has emerged as a promising indicator of dietary quality and cardiovascular risk (7). Several recent studies, including those by Li et al. (8) and Okuda et al. (9), have reported that a higher Na/K ratio is independently associated with elevated BP and adverse cardiovascular outcomes. However, most such evidence originates from general populations or untreated individuals, and its relevance among those receiving antihypertensive therapy remains uncertain (10). Given that antihypertensive medications can strongly influence sodium balance and vascular reactivity, the independent or modifying role of Na/K ratio in treated hypertensive populations may differ substantially from untreated groups (11).

Despite the physiological interplay between sodium, potassium, vascular tone, and endothelial function, the extent to which dietary Na/K ratio may modify antihypertensive drug effectiveness has rarely been investigated. This gap is particularly important because real-world hypertension management relies on both pharmacologic treatment and lifestyle modification, and understanding their interaction is crucial for guiding individualized care. Without such evidence, opportunities to refine treatment strategies based on combined dietary and pharmacologic factors remain limited.

Therefore, we analyzed data from the National Health and Nutrition Examination Survey (NHANES) to evaluate the association between antihypertensive treatment strategies—defined as monotherapy or combination therapy—and two key outcomes: BP control and composite CVD. We further examined whether the dietary Na/K ratio modifies these associations. In addition, to address the lack of mechanistic evidence underlying population-level findings, we supplemented the epidemiological analysis with endothelial cell experiments to explore how high Na/K conditions affect vascular injury and whether antihypertensive drugs retain their protective effects under such conditions. By integrating nationally representative data with mechanistic validation, this study aims to provide a more comprehensive perspective on the interplay between diet, antihypertensive therapy, and cardiovascular health.

## 2 Materials and methods

### 2.1 Study design and data source

NHANES data served as the basis for this cross-sectional investigation, utilizing a multistage survey system intended to monitor the health and dietary patterns of non-institutionalized civilians in the United States (U.S.) NHANES derives its representative dataset from structured interviews, clinical evaluations, and lab measurements. To ensure adequate sample size and representativeness, data from the 2009–2018 cycles were pooled.

Participants aged ≥20 years with a self-reported history of hypertension, available BP measurements, ongoing antihypertensive medication use, and complete 24-hour self-reported dietary intake data on sodium and potassium were eligible for inclusion. Additionally, availability of key covariates—such as body mass index (BMI), smoking status, and chronic disease history—was required.

Exclusion criteria included pregnancy; advanced kidney failure (eGFR < 15 mL/min/1.73 m^2^); implausible dietary energy intake (< 500 or >5000 kcal/day); and missing or biologically implausible BP data. Participants with incomplete data for any primary exposures, outcomes, or essential covariates were also excluded.

**Figure 1:**
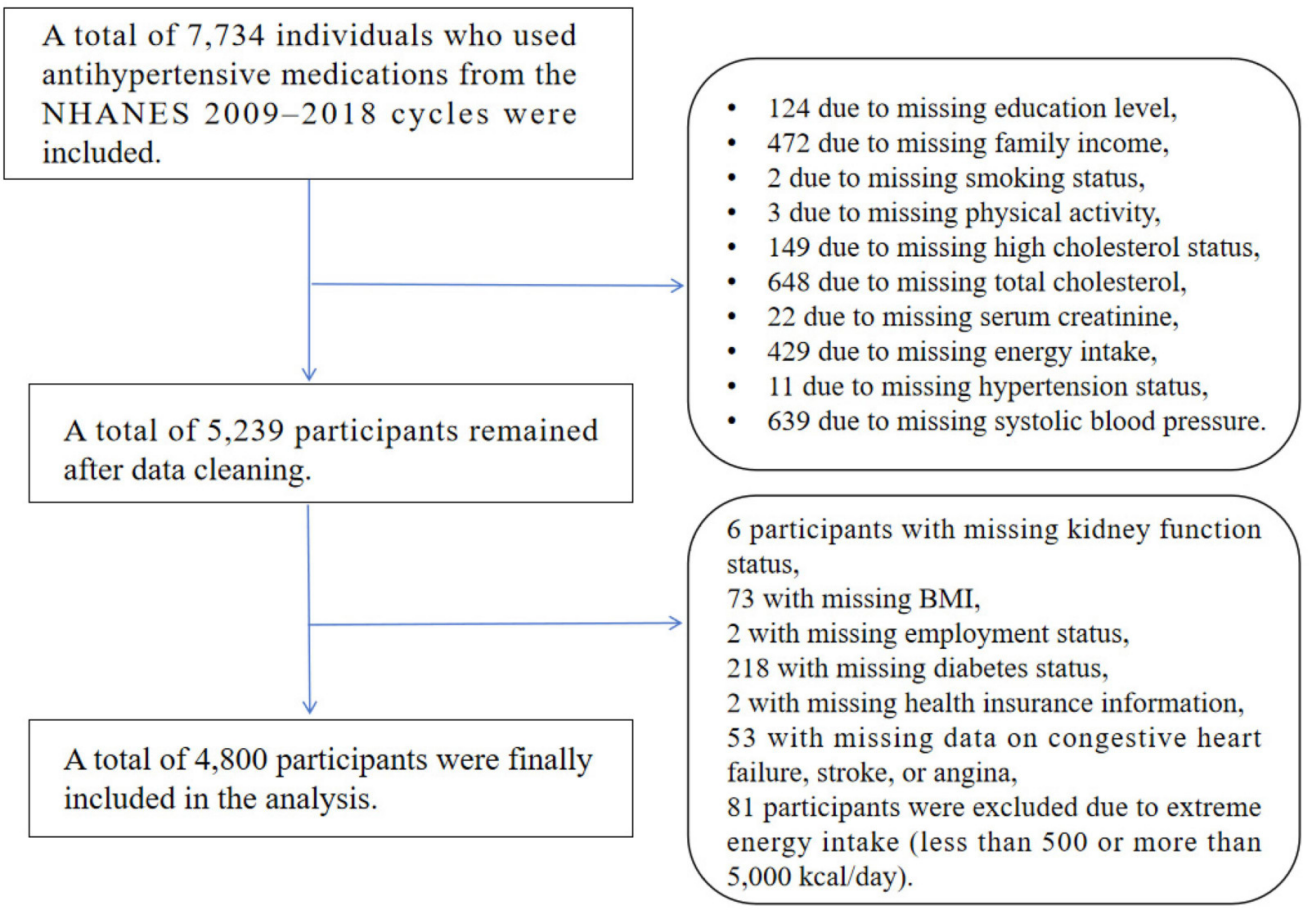
Flowchart of participant selection from NHANES 2009–2018. The diagram outlines the screening process used to identify eligible participants for inclusion. A total of 7,734 individuals who reported using antihypertensive medications were initially identified. After stepwise exclusion based on missing data (demographics, clinical measures, and dietary variables) and implausible energy intake, 4,800 participants with complete information were retained for analysis.

As shown in Figure 1, 7,734 adults using antihypertensive medications were initially identified. Once inclusion and exclusion conditions were applied, 4,800 participants were finalized for analysis. All data were de-identified and publicly available; therefore, institutional review board (IRB) approval and informed consent were not required for this secondary analysis.

### 2.2 Exposure and treatment definition

This study focused on two primary exposures: the antihypertensive treatment strategy and dietary Na/K ratio.

Antihypertensive treatment was defined using self-reported prescription medication use. Participants reporting only one class of antihypertensive drugs were categorized as receiving monotherapy; those using ≥2 different drug classes were classified as receiving combination therapy. Drug classes included aldosterone receptor antagonists, alpha-blockers, angiotensin-converting enzyme inhibitors (ACEIs), angiotensin receptor blockers (ARBs), beta-blockers, calcium channel blockers (CCBs), centrally acting agents (e.g., clonidine), direct vasodilators, diuretics, and renin inhibitors, as per established classifications (12, 13).

The Na/K ratio was calculated from the first 24-hour dietary recall using daily sodium and potassium intakes (mg) from NHANES dietary variables DR1TSODI (sodium) and DR1TKALI (potassium). We prioritized dietary recall data for the primary analysis to maintain a consistent and direct measure of intentional dietary intake, which is the target of public health dietary recommendations. Urinary electrolyte measures, while valuable for assessing physiological balance, were not used as the primary exposure due to their susceptibility to variation from non-dietary factors, and their reflection of systemic homeostasis rather than diet composition itself. The ratio was computed by dividing sodium intake by potassium intake, then categorized into quartiles (Q1–Q4) based on sample distribution.

Both antihypertensive treatment strategy and Na/K ratio were analyzed as independent variables and jointly. Interaction terms were included to assess whether dietary electrolyte balance modified the association between treatment and clinical outcomes.

### 2.3 Outcome measures and covariates

BP control was considered the primary outcome, defined as SBP < 130 mmHg and DBP < 80 mmHg following NHANES standards, with the mean calculated from a maximum of three recordings.

The secondary outcome was the composite prevalence of self-reported physician-diagnosed CVD, defined as a positive history of stroke, myocardial infarction, or heart failure. The outcome thus represents existing CVD status, not incidence.

Covariates included demographics (age, sex, race/ethnicity, education, income, employment, health insurance), health behaviors (smoking status, BMI, physical activity), and clinical history [diabetes, impaired kidney function (eGFR <60 mL/min/1.73 m^2^), dyslipidemia]. Laboratory data such as total cholesterol and dietary energy intake were also included to adjust for potential confounding and reporting bias. Variable definitions followed NHANES coding protocols to ensure consistency.

### 2.4 Model construction

Three hierarchical multivariable logistic regression models were constructed to evaluate the relationships between antihypertensive treatment strategy (monotherapy vs. combination therapy), Na/K ratio (categorized into quartiles), and two primary clinical outcomes: blood pressure (BP) control and cardiovascular disease (CVD) prevalence. Model 1 included only the main exposure variables (treatment strategy and Na/K quartile). Model 2 was adjusted for demographic and behavioral covariates, including age, sex, race/ethnicity, education level, household income, employment status, health insurance coverage, smoking status, BMI, and physical activity. Model 3 further included clinical variables, such as diabetes status, impaired kidney function (eGFR <60 mL/min/1.73 m^2^), total serum cholesterol, and total dietary energy intake. To explore effect modification, interaction on a multiplicative scale was modeled using treatment strategy and Na/K quartiles. Additive effects were quantified using three established indicators: RERI, AP, and S.

In addition to adjusting for gender as a covariate, we formally examined the modifying effect of gender on the outcome by including the multiplicative interaction term between gender and the quartiles of sodium-potassium ratio.

### 2.5 Modeling and grouping

To complement the population-level findings and explore potential mechanisms underlying the associations observed in the NHANES analysis, we established an *in vitro* endothelial injury model using human umbilical vein endothelial cells (HUVECs, ATCC, PCS-100-013). Endothelial dysfunction is a key pathological component in hypertension and cardiovascular disease; therefore, HUVECs were selected to simulate vascular injury under different sodium–potassium conditions. Two culture environments were created: a normal Na/K condition using standard endothelial medium (∼125 mM Na^+^), and a high Na/K condition generated by supplementing the medium with 15 mM NaCl. Angiotensin II (Ang II, 1 μM) was applied to induce hypertension-related endothelial stress. To evaluate drug effects in parallel with clinical treatment strategies, HUVECs were pretreated for 1 hour with either losartan monotherapy (10 μM) or combination therapy consisting of losartan (10 μM) plus amlodipine (5 μM), followed by 24-hour exposure to Ang II.

### 2.6 Cell viability assay

Cell viability was evaluated using a CCK-8 assay (Beyotime, C0037) to quantify metabolic activity after 24 hours of treatment. Following exposure to Ang II and drug interventions under the designated Na/K conditions, CCK-8 working solution was added directly to each well and incubated at 37 ℃ for 1 hour. Absorbance at 450 nm was measured using a microplate reader (BioTek Synergy H1). The resulting optical density values reflected the proportion of viable cells and were used to compare injury severity and drug-mediated protection across groups.

### 2.7 Assessment of intracellular oxidative stress

Intracellular reactive oxygen species (ROS) generation was assessed using the fluorescent probe DCFH-DA (Beyotime, S0033S). After treatments, HUVECs were incubated with 10 μM DCFH-DA at 37 ℃ for 30 minutes in the dark, followed by gentle washing with PBS to remove excess dye. Fluorescence images were captured using a standardized exposure protocol on a fluorescence microscope (Leica DMi8). Semi-quantitative fluorescence intensity was analyzed using ImageJ software to evaluate oxidative stress levels under different Na/K and pharmacologic conditions.

### 2.8 Measurement of endothelial functional markers

Endothelial functional alterations were quantified by measuring nitric oxide (NO) and endothelin-1 (ET-1) in cell culture supernatants. NO production was assessed using a Griess reagent kit (Beyotime, S0021S), which converts accumulated nitrite into a chromophore detectable at 540 nm with a microplate reader. ET-1 concentrations were determined using a commercial ELISA kit (R&D Systems, DET100). These complementary indicators provided an integrated assessment of endothelial vasodilatory and vasoconstrictive function across treatment groups.

### 2.9 Western blot

Protein expression related to endothelial function and inflammation was examined by Western blot. Total protein was extracted using RIPA buffer (Beyotime, P0013B) supplemented with protease inhibitors and quantified via BCA assay (Thermo Fisher, 23227). Equal amounts of protein were separated by SDS-PAGE and transferred onto PVDF membranes (Millipore, IPVH00010). After blocking with 5% non-fat milk, membranes were incubated with primary antibodies against eNOS (Abcam, ab199956), ICAM-1 (Abcam, ab282575), and β-actin (Abcam, ab6276), followed by HRP-conjugated secondary antibodies (Cell Signaling Technology, 7074/7076). Bands were visualized using enhanced chemiluminescence (Thermo Fisher, 32106) and quantified with ImageJ. Protein levels were normalized to β-actin to control for loading variations.

### 2.10 Statistical analysis

Statistical analyses for the NHANES dataset were conducted in R (version 4.3.3), incorporating sampling weights, primary sampling units (PSUs), and strata to account for the complex survey design. Continuous variables were reported as weighted means ± standard errors, categorical variables as weighted percentages, and group differences were assessed using Student's t-tests or chi-square tests as appropriate. Multivariable logistic regression models were used to estimate adjusted odds ratios (ORs) and 95% confidence intervals (CIs), with significance defined as *P* < 0.05. For the mechanistic cell experiments, data from at least three independent replicates were analyzed using SPSS 26.0 and expressed as mean ± standard deviation. Group comparisons were performed using one-way ANOVA followed by Bonferroni correction, with *P* < 0.05 considered statistically significant.

## 3 Result

### 3.1 Baseline characteristics of participants by antihypertensive treatment strategy

As shown in Table 1, participants receiving combination therapy were generally older, with a relatively larger group of individuals aged ≥ 65 years (59.2% vs. 46.5%, *P* < 0.001), and were more likely to be male (51.8% vs. 48.3%, *P* = 0.020) than those on monotherapy. Racial/ethnic composition also differed significantly, with a higher proportion of non-Hispanic individuals in the combination therapy group (75.6% vs. 68.7%, *P* < 0.001).

**Table 1:** Baseline characteristics of participants by antihypertensive treatment strategy.

Characteristic	Category	Single drug	Multiple drugs	*χ* ^2^	*P*
Age, n (%)	<65	1,534 (53.5)	791 (40.8)	77.73	<0.001
	≥65	1,330 (46.5)	1,145 (59.2)		
Gender, n (%)	Male	1,386 (48.3)	1,004 (51.8)	5.41	0.020
	Female	1,478 (51.7)	932 (48.2)		
Race Ethnicity, n (%)	Hispanic	599 (20.9)	328 (16.9)	27.95	<0.001
	Non-Hispanic	1,968 (68.7)	1,464 (75.6)		
	Multi-ethnic	297 (10.4)	144 (7.5)		
Education Level, n (%)	High school education or below	1,382 (48.2)	1,020 (52.7)	8.9	0.003
	College education or above	1,482 (51.8)	916 (47.3)		
Family Income, n (%)	≤20,000	768 (26.8)	535 (27.6)	0.35	0.553
	>20,000	2,096 (73.2)	1,401 (72.4)		
Employment Status, n (%)	No	1,748 (61.0)	1,408 (72.7)	69.63	<0.001
	Yes	1,116 (39.0)	528 (27.3)		
Health Insurance, n (%)	No	2,618 (91.4)	1,794 (92.7)	2.28	0.131
	Yes	246 (8.6)	142 (7.3)		
Smoking Status, n (%)	No	1,372 (47.9)	1,032 (53.3)	13.26	<0.001
	Yes	1,492 (52.1)	904 (46.7)		
BMI, n (%)	<30	1,507 (52.6)	873 (45.1)	25.87	<0.001
	≥30	1,357 (47.4)	1,063 (54.9)		
Physical Activity, n (%)	No	443 (15.5)	285 (14.7)	0.44	0.505
	Yes	2,421 (84.5)	1,651 (85.3)		
Diabetes Diagnosis, n (%)	No	851 (29.7)	776 (40.1)	54.97	<0.001
	Yes	2,013 (70.3)	1,160 (59.9)		
Kidney Disease, n (%)	No	154 (5.4)	226 (11.7)	61.96	<0.001
	Yes	2,710 (94.6)	1,710 (88.3)		
High Cholesterol, n (%)	No	1,650 (57.6)	1,249 (64.5)	22.72	<0.001
	Yes	1,214 (42.4)	687 (35.5)		
Energy Intake (kcal), n (%)	Low	1,397 (48.8)	1,002 (51.8)	3.98	0.046
	High	1,467 (51.2)	934 (48.2)		
Total Cholesterol, n (%)	<6.22	2,556 (89.2)	1,781 (92.0)	9.7	0.002
	≥6.22	308 (10.8)	155 (8.0)		

Participants in the combination group had a higher likelihood of lower educational attainment (52.7% vs. 48.2%, *P* = 0.003) and unemployment (72.7% vs. 61.0%, *P* < 0.001). No significant group differences were found in family income, physical activity, or health insurance coverage (all *P* > 0.05).

Clinically, the combination therapy group exhibited a greater prevalence of obesity (54.9% vs. 47.4%, *P* < 0.001), but showed a reduced prevalence of diabetes (59.9% vs. 70.3%, *P* < 0.001), kidney disease (88.3% vs. 94.6%, *P* < 0.001), self-reported high cholesterol (35.5% vs. 42.4%, *P* < 0.001), and elevated total cholesterol (≥6.22 mmol/L: 8.0% vs. 10.8%, *P* = 0.002) relative to the monotherapy group. They also had a slightly higher non-smoking rate (53.3% vs. 47.9%, *P* < 0.001) and lower overall dietary energy intake (51.8% vs. 48.8%, *P* = 0.046).

### 3.2 Association between treatment strategy and clinical outcomes

**Figure 2:**
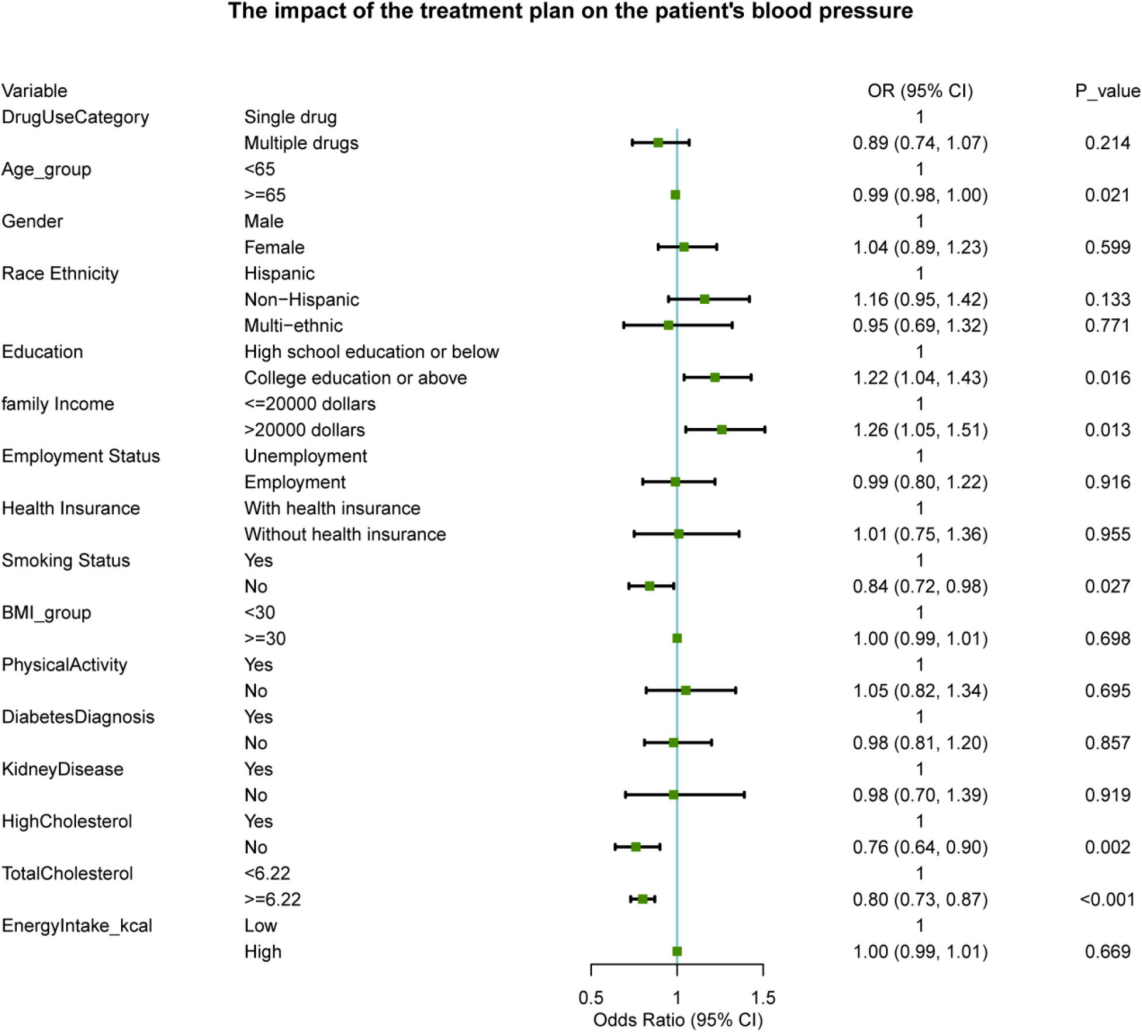
Multivariable logistic regression: association between antihypertensive treatment and blood pressure control.

As shown in Figure 2, after correcting for confounding factors, several variables were significantly associated with the likelihood of achieving blood pressure control. The non-smoking status (OR = 0.84, 95% CI: 0.72–0.98, *P* = 0.027) and the absence of high cholesterol (OR = 0.76, 95% CI: 0.64 - 0.90, *P* = 0.002) were associated with better blood pressure control. Age ≥65 years (OR = 0.99, 95% CI: 0.98 - 1.00, *P* = 0.021) was marginally associated with poor blood pressure control. It is notable that there was no significant association between antihypertensive treatment strategies (combination therapy vs. single therapy) and the likelihood of blood pressure control (OR = 0.89, 95% CI: 0.74 - 1.07, *P* = 0.214).

**Figure 3:**
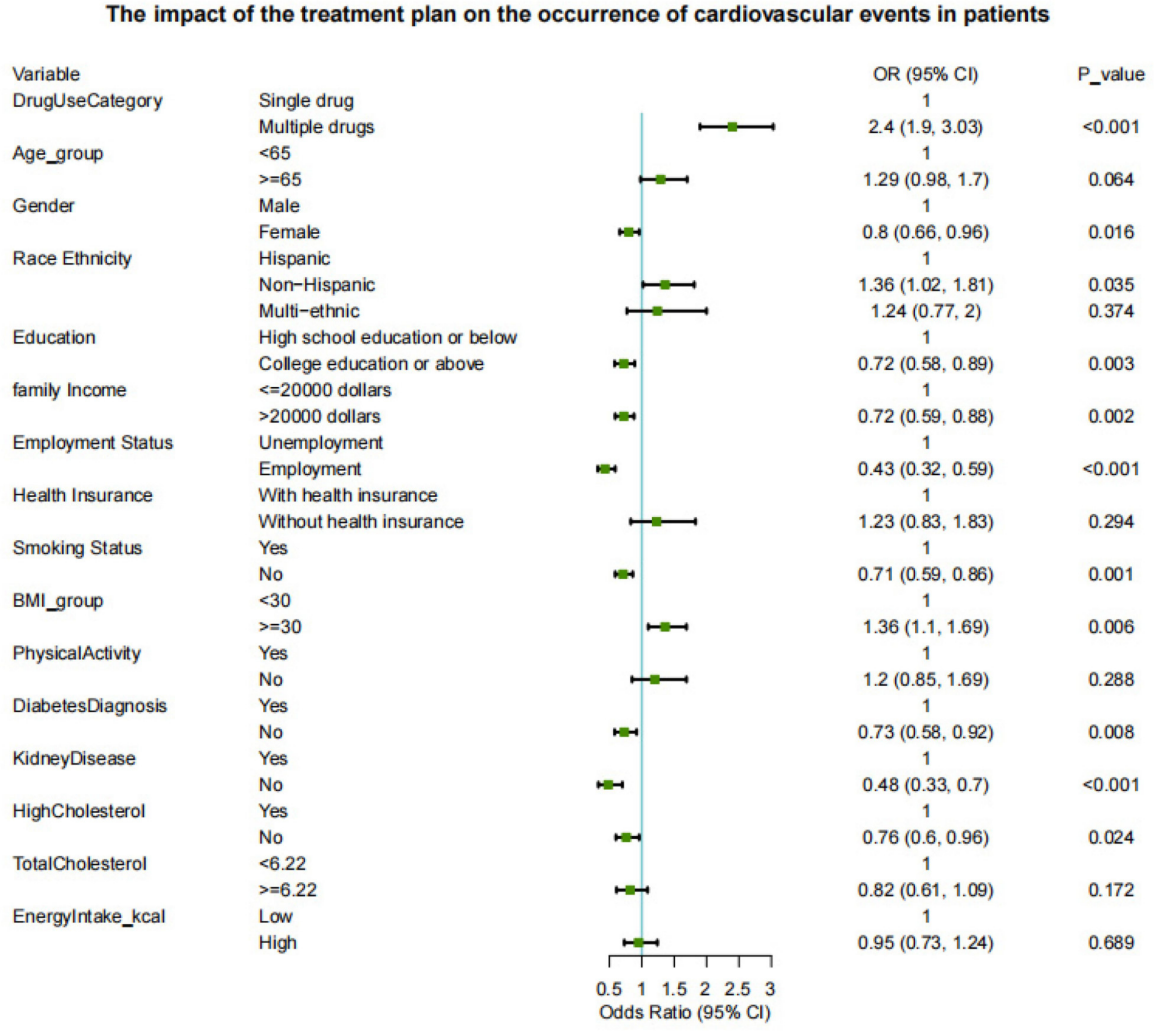
Multivariable logistic regression: association between antihypertensive treatment and composite CVD.

In Figure 3, combination therapy was associated with a substantially increased likelihood of composite CVD (OR = 2.40, 95% CI: 1.90–3.03, *P* < 0.001). Other risk factors included non-Hispanic ethnicity (OR = 1.36, *P* = 0.035) and BMI ≥ 30 (OR = 1.36, *P* = 0.006). Conversely, cardiovascular risk was significantly lower among women, those with higher education, greater income, and employment, as well as non-smokers, and individuals without diabetes, kidney disease, or hypercholesterolemia (all *P* < 0.05).

### 3.3 Association between sodium-to-potassium ratio and clinical outcomes

**Figure 4:**
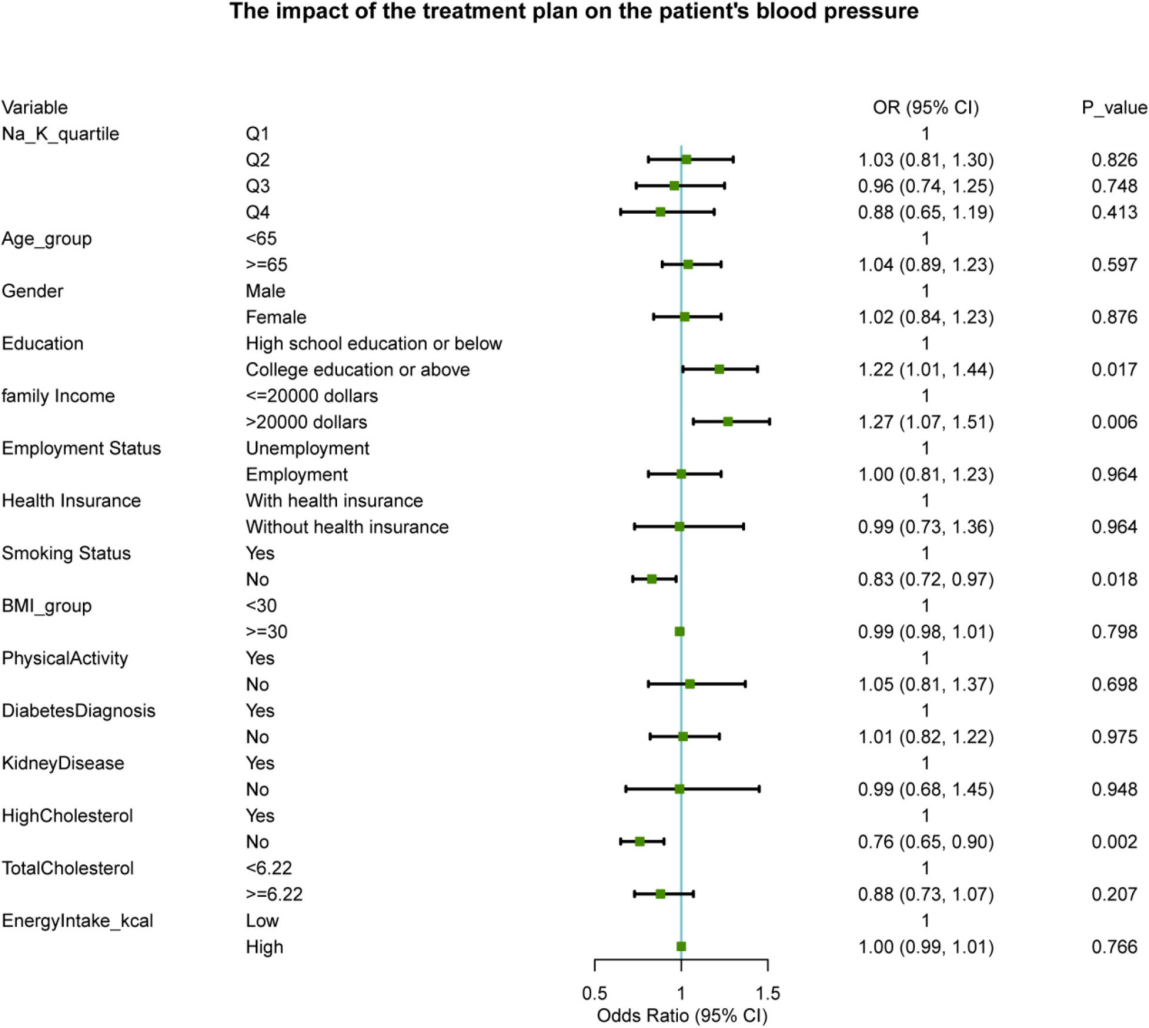
Association between dietary sodium-to-potassium ratio and blood pressure control.

As shown in Figure 4, multivariate logistic regression analysis revealed that after adjusting for demographic, socioeconomic, behavioral, and clinical covariates, the dietary Na/K ratio was not significantly associated with blood pressure control. Specifically, compared to the lowest quartile (Q1), the dietary Na/K ratio was not significantly associated with the likelihood of achieving blood pressure control, even in the highest quartile (Q4) (OR = 0.88, 95% CI: 0.65-1.19, *P* = 0.413). In contrast, not being a smoker (OR = 0.83, 95% CI: 0.72-0.97, *P* = 0.018) and having no high cholesterol (OR = 0.76, 95% CI: 0.65-0.90, *P* = 0.002) were associated with better blood pressure control.

**Figure 5:**
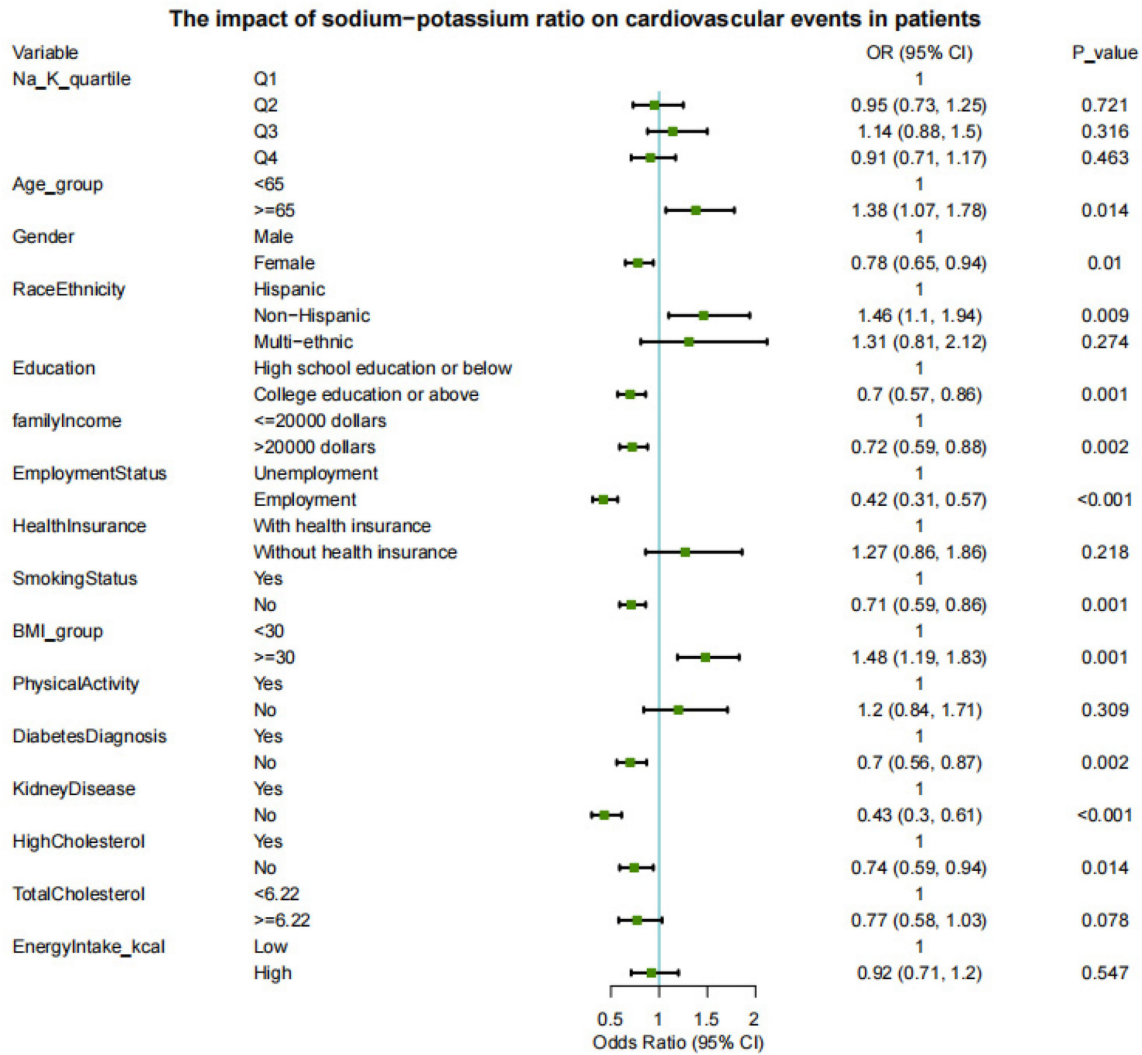
Association between dietary sodium-to-potassium ratio and composite CVD.

Similarly, the Na/K ratio did not demonstrate a significant association with composite CVD across all quartiles (all *P* > 0.05) (Figure 5). In contrast, several covariates remained significantly associated with cardiovascular outcomes. Risk factors included age ≥ 65 years (OR = 1.38, *P* = 0.014), obesity (BMI ≥ 30; OR = 1.48, *P* = 0.001), and non-Hispanic ethnicity (OR = 1.46, *P* = 0.009). Conversely, protective factors included female sex (OR = 0.78, *P* = 0.010), higher education (OR = 0.70, *P* = 0.001), higher household income (> 20,000 USD; OR = 0.72, *P* = 0.002), employment (OR = 0.42, *P* < 0.001), non-smoking status (OR = 0.71, *P* = 0.001), and absence of kidney disease (OR = 0.74, *P* = 0.014). In addition, individuals without diabetes (OR = 0.70, *P* = 0.002) and those without high cholesterol (OR = 0.74, *P* = 0.014) also had significantly reduced cardiovascular risk. Total cholesterol level, however, was not independently associated with composite CVD (*P* = 0.078).

### 3.4 Interaction analysis between treatment strategy and sodium-to-potassium ratio

Table 2 shows that in terms of BP control, the ORs of each group were close to 1 compared with the reference group (single drug + low sodium-potassium ratio), and the *P*-values were all >0.05. This indicates that in terms of BP control, there is no significant individual effect or interaction between drug use and sodium-potassium ratio. In line with this, additive interaction metrics [RERI, AP, and synergy index (S)] were non-significant, and their 95% confidence intervals all spanned 0 or 1, indicating no evidence of statistical interaction. For CVD outcomes, a different pattern emerged. While monotherapy with a high Na/K ratio was not associated with increased risk (OR = 0.82, *P* = 0.3883), combination therapy—regardless of Na/K level—was associated with significantly elevated prevalent CVD: OR = 1.93 for low Na/K (*P* < 0.001) and OR = 2.51 for high Na/K (*P* < 0.001). Additive interaction metrics (RERI = 0.764, AP = 0.304, S = 2.02) were all positive but not statistically significant, as confidence intervals included the null, suggesting a possible but inconclusive trend toward interaction.

**Table 2:** Interaction between antihypertensive treatment and sodium-to-potassium ratio on blood pressure control and composite CVD.

Treatment Strategy	Na/K Ratio	Blood Pressure Control	CVD
		OR (95% CI), P	OR (95% CI), P
Monotherapy	Low	REF	REF
Monotherapy	High	1.14 (0.88–1.47), 0.5163	0.82 (0.65–1.04), 0.3883
Combination therapy	Low	1.17 (0.87–1.58), 0.4605	1.93 (1.14–3.28), < 0.001
Combination therapy	High	1.06 (0.82–1.38), 0.92	2.51 (1.19–5.29), < 0.001
RERI 95%CI		−0.257 (−0.789–0.275)	0.764 (−1.374–2.902)
AP 95%CI		−0.243 (−0.783–0.296)	0.304 (−0.359 −0.967)
S 95%CI		0.181 (−0.737 −1.099)	2.021 (−1.737 −5.779)

Stratified analyses in Table 3 further demonstrated that combination therapy consistently increased prevalent CVD across all Na/K quartiles, with ORs ranging from 1.61 to 3.93, all *P* < 0.05, but no significant interaction emerged between Na/K ratio and treatment strategy (P for interaction > 0.05 in all quartiles), supporting an independent and additive effect of each factor. In Table 4, no significant association was found between treatment strategy and BP control within any Na/K stratum, and no interaction was revealed (*P* > 0.3 across all quartiles).

**Table 3:** Effects of sodium-potassium ratio quartiles on treatment regimens on composite CVD.

sodium-potassium ratio	Drug Use Category	OR (95% CI)	*P*	*P* for interaction
Quartile 1	Single drug	ref		0.86
	Multiple drugs	2.40 (1.64, 3.53)	<0.001	
Quartile 2	Single drug	ref		0.106
	Multiple drugs	1.61 (1.11, 2.33)	0.014	
Quartile 3	Single drug	ref		0.58
	Multiple drugs	2.74 (1.66,4.53)	<0.001	
Quartile 4	Single drug	ref		0.279
	Multiple drugs	3.93 (2.42,6.37)	<0.001	

**Table 4:** Effects of sodium-potassium ratio quartiles on treatment regimens on blood pressure control.

sodium-potassium ratio	Drug Use Category	OR (95% CI)	*P*	*P* for interaction
Quartile 1	Single drug	ref		0.704
	Multiple drugs	1.34 (0.92–1.95)	0.130	
Quartile 2	Single drug	ref		0.319
	Multiple drugs	1.07 (0.72–1.58)	0.744	
Quartile 3	Single drug	ref		0.697
	Multiple drugs	0.85 (0.59–1.23)	0.384	
Quartile 4	Single drug	ref		0.633
	Multiple drugs	1.08 (0.75–1.55)	0.675	

### 3.5 Effect modification by sex

We formally tested the moderating effect of the interaction between gender and the sodium-potassium ratio by introducing a multiplicative interaction term. For the association between the sodium/potassium ratio in the diet and blood pressure control, we observed a significant interaction effect at the moderate sodium/potassium level (Table 5). Specifically, the interaction term between sodium/potassium ratio Q2 and females (OR = 1.83, 95% CI: 1.23-2.73, *P* = 0.003) and the interaction term between Q3 and females (OR = 1.65, 95% CI: 1.18-2.29, *P* = 0.003) were statistically significant, indicating that within these quartiles, the association between the sodium/potassium ratio and blood pressure control differed between males and females. For the interaction term between Q4 and females (*P* = 0.389), no significant interaction was observed.

**Table 5:** Interaction test between gender and sodium-potassium ratio.

Variable	Comparison	OR (95% CI)	*P*-value
Main Effects			
Na/K ratio quartile	Q2 vs Q1	0.74 (0.51, 1.08)	0.123
Na/K ratio quartile	Q3 vs Q1	0.91 (0.61, 1.36)	0.648
Na/K ratio quartile	Q4 vs Q1	0.80 (0.57, 1.14)	0.231
Sex	Female vs male	0.84 (0.62, 1.13)	0.239
Interaction Terms			
Na/K ratio quartile × sex	Interaction: Q2 × female	1.83 (1.23, 2.73)	0.003
Na/K ratio quartile × sex	Interaction: Q3 × female	1.65 (1.18, 2.29)	0.003
Na/K ratio quartile × sex	Interaction: Q4 × female	1.17 (0.81, 1.70)	0.389

### 3.6 Association between sodium-to-potassium ratio and clinical outcomes based on quartile analysis

To evaluate the association between Na/K ratio and clinical outcomes, participants were distributed among quartiles, with Quartile 1 (Q1) serving as the reference group. As shown in Table 6, across all three models—including the fully adjusted model (Model 3)—there was no statistically significant association between Na/K quartiles and blood pressure control. The ORs for Quartiles 2 to 4 were 0.95 (95% CI: 0.73–1.25, *P* = 0.721), 1.14 (95% CI: 0.88–1.50, *P* = 0.316), and 0.91 (95% CI: 0.71–1.17, *P* = 0.463), respectively, indicating no clear trend or dose–response pattern.

**Table 6:** Effect of sodium-potassium ratio on blood pressure control according to quartile analysis.

Na/K Ratio Quartile	Crude Model (Model 1)	Partially Adjusted Model (Model 2)	Fully Adjusted Model (Model 3)
	OR (95% CI), P	OR (95% CI), P	OR (95% CI), P
Quartile 1	Reference	Reference	Reference
Quartile 2	0.93 (0.71–1.22), 0.598	0.95 (0.73–1.24), 0.695	0.95 (0.73–1.25), 0.721
Quartile 3	1.05 (0.82–1.35), 0.692	1.12 (0.85–1.46), 0.417	1.14 (0.88–1.50), 0.316
Quartile 4	0.94 (0.73–1.21), 0.634	0.96 (0.75–1.23), 0.759	0.91 (0.71–1.17), 0.463

Similarly, Table 7 presents the association between Na/K quartiles and composite CVD. In the fully adjusted model, the odds ratios were 1.16 (95% CI: 0.92–1.47, *P* = 0.193) for Q2, 1.05 (95% CI: 0.83–1.34, *P* = 0.661) for Q3, and 1.22 (95% CI: 0.94–1.59, *P* = 0.132) for Q4. Although a modest upward trend in risk estimates was observed across quartiles, particularly in Q4, none reached statistical significance, and all effect estimates remained close to the null (OR ≈ 1.0).

**Table 7:** Effect of sodium-potassium ratio on composite CVD according to quartile analysis.

Na/K Ratio Quartile	Crude Model (Model 1)	Partially Adjusted Model (Model 2)	Fully Adjusted Model (Model 3)
	OR (95% CI), P	OR (95% CI), P	OR (95% CI), P
Quartile 1	Reference	Reference	Reference
Quartile 2	1.11 (0.88–1.39), 0.365	1.16 (0.92–1.45), 0.201	1.16 (0.92–1.47), 0.193
Quartile 3	0.96 (0.75–1.21), 0.708	1.04 (0.82–1.32), 0.741	1.05 (0.83–1.34), 0.661
Quartile 4	1.04 (0.82–1.32), 0.742	1.20 (0.92–1.55), 0.172	1.22 (0.94–1.59), 0.132

Collectively, a higher sodium-to-potassium intake ratio appears unrelated to blood pressure regulation or composite CVD when considered independently in hypertensive subjects.

### 3.7 Effects of Na/K conditions and antihypertensive treatments on endothelial injury

**Figure 6:**
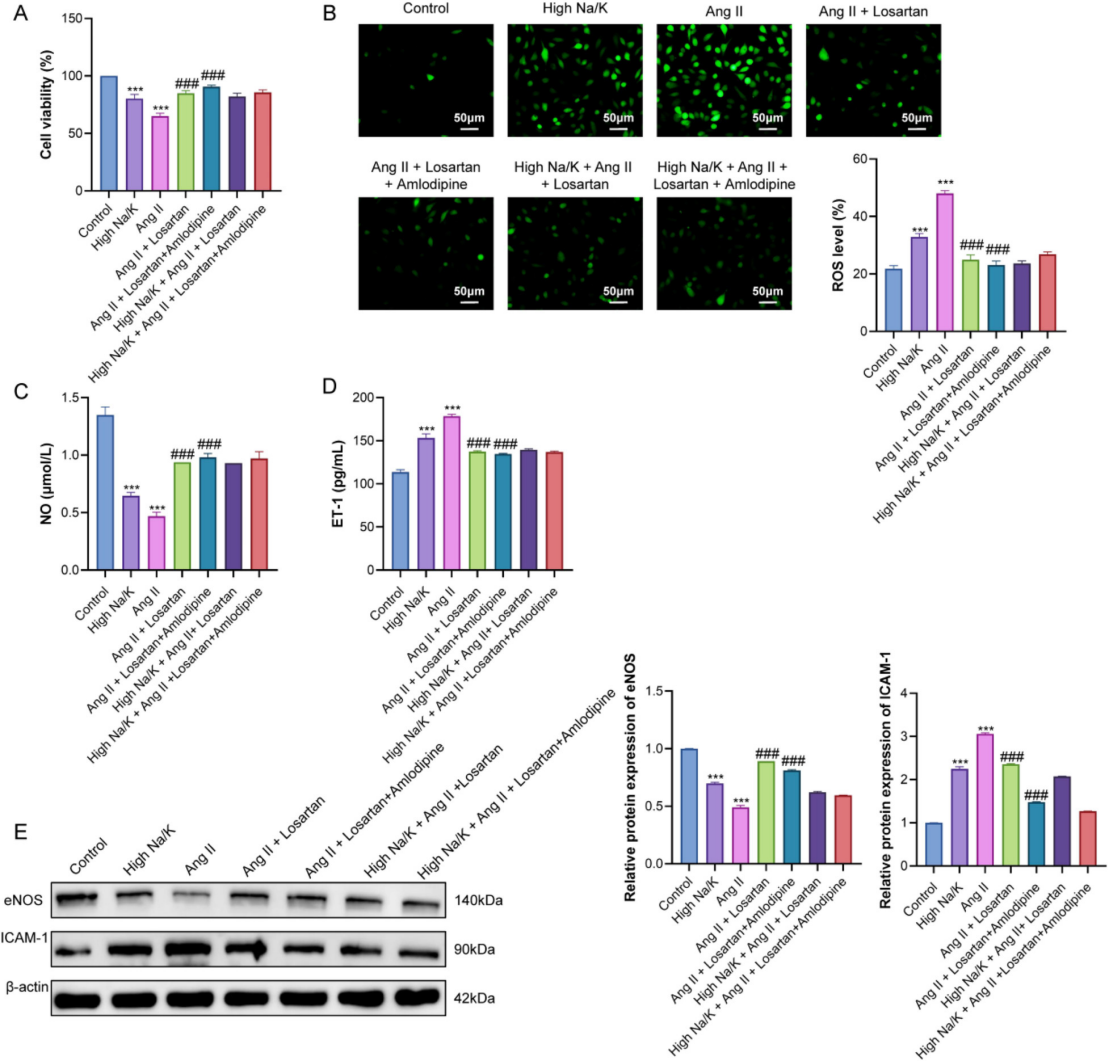
Effects of Na/K conditions and antihypertensive treatments on endothelial injury in HUVECs. **(A)** Cell viability assessed by CCK-8. **(B)** Intracellular ROS detected by DCFH-DA fluorescence. **(C)** NO levels determined by Griess assay. **(D)** ET-1 levels determined by ELISA. **(E)** Western blot analysis of eNOS and ICAM-1 expression; β-actin served as loading control. ***P < 0.001 vs. Control group. ###*P* < 0.001 vs. Ang II group.

HUVEC viability showed a clear response to Na/K conditions and Ang II stimulation (Figure 6A). High Na/K reduced cell viability relative to control (*P* < 0.001), and Ang II further decreased viability (*P* < 0.001). Under normal Na/K conditions, losartan and combination therapy both increased viability compared with Ang II alone (*P* < 0.001). In high Na/K medium, viability after drug treatment was slightly lower than under normal Na/K, but the differences were not statistically significant (*P* > 0.05).

Intracellular ROS levels increased markedly under high Na/K and Ang II exposure (both *P* < 0.001). Drug treatment reduced ROS accumulation under normal Na/K (*P* < 0.001). In high Na/K conditions, ROS remained elevated compared with the corresponding normal Na/K groups, although differences did not reach statistical significance (*P* > 0.05) (Figure 6B).

Analysis of endothelial functional markers showed reduced NO production in both high Na/K and Ang II groups compared with control (*P* < 0.05) (Figure 6C). Losartan and combination therapy increased NO levels relative to Ang II (*P* < 0.001). Under high Na/K, NO values after drug treatment were slightly lower than in normal Na/K, without significant differences (*P* > 0.05). ET-1 exhibited an opposite pattern, with high Na/K and Ang II significantly increasing ET-1 secretion (*P* < 0.001), and both drug treatments lowering ET-1 relative to Ang II (*P* < 0.001). High Na/K modestly increased ET-1 compared with normal Na/K under the same drug treatments (*P* > 0.05).

Western blot analysis showed decreased eNOS and increased ICAM-1 expression in high Na/K and Ang II groups (*P* < 0.001) (Figure 6D). Drug treatments elevated eNOS and reduced ICAM-1 compared with Ang II (*P* < 0.001). Under high Na/K, eNOS and ICAM-1 levels after drug treatment were close to those observed under normal Na/K, with no statistically significant differences (*P* > 0.05).

Western blot analysis further confirmed the effects of Na/K conditions and antihypertensive treatments on endothelial functional proteins (Figure 6E). High Na/K and Ang II exposure significantly reduced eNOS expression while increasing ICAM-1 levels compared with the control group (both *P* < 0.001), indicating endothelial dysfunction and enhanced inflammatory activation. Treatment with losartan or combination therapy partially restored eNOS expression and lowered ICAM-1 relative to Ang II alone (both *P* < 0.001). Notably, under high Na/K conditions, the magnitude of eNOS recovery and ICAM-1 suppression was comparable to that observed under normal Na/K, and differences between the two sodium conditions did not reach statistical significance (*P* > 0.05). These mechanistic findings parallel the NHANES results, indicating that high Na/K exacerbates endothelial injury but does not substantially modify drug-mediated protection.

## 4 Discussion

In this nationally representative cross-sectional study of treated hypertensive adults from NHANES, we evaluated the associations between antihypertensive treatment strategy, dietary sodium-to-potassium (Na/K) ratio, blood pressure control defined using the contemporary <130/80 mmHg threshold, and prevalent cardiovascular disease (CVD), and further explored whether dietary Na/K modified these associations. Our analysis yielded several key observations.

Firstly, the intensity of drug treatment (combination therapy versus single therapy) was not associated with the probability of achieving strict blood pressure control. This suggests that simply adding more drug classes may not overcome the multifaceted barriers to optimal BP control in complex patients. Such patients often suffer from more severe or more resistant hypertension, and have a more severe burden of complications (14, 15). Secondly, combination therapy is positively correlated with the prevalence of cardiovascular diseases (CVD). This association may reflect a clear indication confusion, as individuals requiring multiple drug treatments typically have higher baseline cardiovascular risk characteristics, including more severe comorbidities, such as chronic kidney disease (16). Moreover, the multiple use of medications itself may introduce management complexity, potential drug interactions, or adherence issues, leading to adverse consequences, which has been confirmed in trials evaluating drug reduction strategies (17). Importantly, this difference in treatment intensity and blood pressure control highlights the necessity of incorporating effective lifestyle adjustments and metabolic risk reduction into management plans, rather than relying solely on intensified drug treatment. Thirdly, there is no significant independent association between the sodium/potassium ratio in the diet and blood pressure control or common cardiovascular diseases (CVD). It also does not significantly alter the relationship between treatment strategies and these outcomes. This contrasts with evidence from general populations or untreated populations, where a higher sodium/potassium ratio significantly predicts elevated blood pressure and cardiovascular risk (18–21). This difference may indicate that the influence of dietary electrolytes is masked by the drug-mediated vascular tension and sodium balance regulation in individuals receiving drug treatment (10, 22, 23). Importantly, our endothelial cell experiments provide a direct mechanistic explanation for the result found in the NHANES cohort study: under high sodium/potassium conditions, angiotensin II-induced oxidative stress and endothelial dysfunction are exacerbated, while the protective effect of losartan (and combination therapy) remains unchanged, which is consistent with previous research results (24, 25). This indicates that despite the adverse dietary electrolyte imbalance, the pharmacological effects of these antihypertensive drugs still have strong efficacy. Therefore, from a biological perspective, changes in sodium and potassium intake in the diet of patients receiving treatment are unlikely to significantly alter the efficacy of the drugs in clinical indicators, because the core protective mechanism of the drugs remains unchanged. This direct translation from the laboratory to the population strengthens the conclusion that in patients receiving drug treatment, the influence of the sodium and potassium ratio in the diet is effectively counteracted by the effective drug treatment, thus shifting the clinical focus to optimizing the drug regimen and managing other modifiable risk factors. Fourthly, by further expanding our main analysis, we formally tested and found that gender significantly alters the association between the dietary sodium/potassium ratio and blood pressure control (the interaction *P*-value for Q2 and Q3 is 0.003). This indicates that men and women have different physiological responses or sensitivities to dietary electrolytes, which may be related to sodium sensitivity differences or the effects of hormones on vascular function. This finding emphasizes the importance of considering gender as a confounding factor in nutritional epidemiology and supports the pursuit of more personalized dietary guidance.

Nonetheless, several limitations merit consideration. The cross-sectional nature of NHANES precludes causal inference, establishes no temporality, and cannot establish causal mediation pathways, while self-reported CVD events may introduce recall bias. Dietary Na/K ratios were derived from a single 24-hour recall, which is subject to within-person variability and may introduce regression dilution bias, thereby attenuating observed associations toward the null. It should also be noted that we relied on self-reported dietary intake rather than urinary electrolyte measures. Although urinary data provide a more objective biomarker, they reflect integrated physiological balance rather than diet composition itself. Future studies incorporating both dietary and urinary assessments would offer a more comprehensive evaluation. Furthermore, data on key clinical details such as duration of hypertension, exact drug dosage, and medication adherence were unavailable, leaving potential for residual confounding despite adjustment for a wide range of covariates. Additionally, detailed data on alcohol consumption patterns were not available for model inclusion; this may represent a source of residual confounding given alcohol's potential influence on electrolyte homeostasis and cardiovascular risk. Comprehensive covariate adjustment was performed; however, unmeasured residual confounding, including genetic and stress-related factors, cannot be entirely ruled out. Future research should focus on prospective cohort studies or randomized controlled trials incorporating more precise dietary and pharmacologic assessments, particularly among high-risk subgroups such as older adults or patients with metabolic syndrome.

## 5 Conclusion

Combination therapy was associated with higher prevalence of CVD without improving blood pressure control. The dietary sodium-to-potassium ratio showed no independent effect and did not modify treatment outcomes. Cell experiments confirmed that high Na/K aggravated endothelial injury, while drug-mediated protection remained similar across sodium conditions. Moreover, the absence of significant dietary Na/K effect modification in the population cohort is mechanistically explained by the preserved drug efficacy under high Na/K stress. These findings suggest that pharmacologic treatment intensity and dietary electrolyte balance act independently in treated hypertension.

## Data Availability

The original contributions presented in the study are included in the article/Supplementary Material, further inquiries can be directed to the corresponding author.
